# Characteristics of tropical human-modified forests after 20 years of natural regeneration

**DOI:** 10.1186/s40529-017-0190-x

**Published:** 2017-08-30

**Authors:** Lih-Chyun Loo, Guo-Zhang M. Song, Kuo-Jung Chao

**Affiliations:** 1 International Master Program of Agriculture, National Chung Hsing University, 145 Xingda Road, South District, Taichung, 40227 Taiwan; 2 Department of Soil and Water Conservation, National Chung Hsing University, 145 Xingda Road, South District, Taichung, 40227 Taiwan

**Keywords:** Arboretum, Forest composition, Forest structure, Plantation, Regeneration, Secondary forest, Species diversity refuge

## Abstract

**Background:**

Abandoned human-modified forests are refuges for remnant biodiversity. However, there are very few studies on the biodiversity and regeneration of native species in human-modified forests which are rich in exotic trees. Our research aim is to evaluate the regeneration status and biodiversity of two adjacent human-modified forests. The two forests have distinct overstorey exotic species richness prior to abandonment: one is an exotic tree plantation low in species richness, and the other is an exotic arboretum high in species richness. The original management practices of the two forests have been neglected for more than 20 years. A primary forest was selected as a reference forest to compare their diversity and regeneration status. We asked: (1) Is there a structural difference among the three forests? (2) What are the proportions of native saplings in the human-modified forests? (3) Are the introduced exotic species able to naturalize?

**Results:**

We recorded 1316 individuals from 88 species, belonging to 69 genera and 34 families in the three forests [each sampled 16 quadrats (10 m × 5 m)]. Both human-modified forests were similar in their height structure, diameter structure, and sapling density, but differed in species diversity (characterized by rarefaction curves) and floristic composition (indicated by a quantitative similarity index). In the arboretum, only 50% of the sapling individuals were native. Surprisingly, when sampling efforts were standardized, the arboretum had higher native sapling species richness than the exotic species-poor plantation. Moreover, both human-modified forests had conserved a few rare and endemic species. Nevertheless, some exotic species in the arboretum had escaped to the nearby plantation.

**Conclusions:**

After 20 years of abandonment, the two human-modified forests had converged in structure, but not in diversity patterns of native saplings. This could be due to that the diversity of exotic overstorey composition can influence the natural regeneration of understorey plants. Our study also raised concerns about conserving native species and managing naturalized exotic species in these human-modified forests.

**Electronic supplementary material:**

The online version of this article (doi:10.1186/s40529-017-0190-x) contains supplementary material, which is available to authorized users.

## Background

Conserving tropical biodiversity cannot neglect the ecological impacts of human-modified forests (Chazdon et al. 2009), due to its ever-increasing areas (Turner and Corlett 1996). Their impacts could be both positive and negative. For example, some exotic (non-native) plantations can be refuges for native biodiversity (Senbeta et al. 2002; Barlow et al. 2007; Bhagwat et al. 2008), when having naturally regenerated native saplings (Senbeta et al. 2002). On the contrary, the introduced exotic species may become invasive and result in negative impacts to native forests (Thijs et al. 2014; Macfarlane et al. 2015).

The key step for assessing the ecological impacts of human-modified forests is by understanding the status of their regeneration. Forest structure and species composition have been suggested as good indicators (Kimmins 2004; Ruiz-Jáen and Aide 2005), as these are commonly collected data in forest assessments. Thus, they can be comparable indicators for diagnosing regeneration status among forests (Ochoa-Gaona et al. 2010; Winter 2012).

Previous studies have demonstrated that human-modified forests have fast structural recovery but slow composition recovery (Zimmerman et al. 1995; Marcano-Vega et al. 2002; Poorter et al. 2016). The regeneration of native saplings under plantation seemed to be varied between the overstorey exotic trees. For example, some exotic overstorey trees (e.g., *Casuarina equisetifolia*) may inhibit the regeneration of native saplings, whereas some (e.g., *Leucaena leucocephala*) may allow it (Parrotta 1995). However, most studies have focused on plantations with monoculture or low diversity of exotic tree species (e.g., coffee or *Eucalyptus* spp.) (Zimmerman et al. 1995; Marcano-Vega et al. 2002; Barlow et al. 2007; Bhagwat et al. 2008). Not many have monitored human-modified forests rich in exotic tree diversity (Bhagwat et al. 2008). It is not clear how the initial overstorey exotic composition may alter the forest regeneration trajectory. Do native species have a chance to come inside a forest rich in exotic species when available habitats might have been occupied by those non-native ones? Moreover, the tens-rule of introduced species predicted that 10% of the introduced species would be a casual alien, 1% of the introduced species would be naturalized, and 0.1% of the introduced species would become invasive (Williamson et al. 1986; Richardson and Pyšek 2006). Thus, a plantation rich in exotic species has some potential to introduce invasive species when human management practices are absent.

Taiwan offers an opportunity to assess the regeneration status of lowland tropical human-modified forests. The island has one of the highest population densities in the world (649 person km^−2^; Taiwan Ministry of the Interior (2015)) and is no exception to the loss of its primary forests (Taiwan Forestry Bureau 2011). The original lowland tropical primary forests were located along the foothills and valleys of the Central Mountain Range (elevation below 500 m) (Su 1984). However, they have been mostly developed as agricultural lands and tree plantations (Editorial Committee of the Flora of Taiwan 1994–2003). Only 0.5% (8507.18 ha) of Taiwan’s currently forested area can be classified as lowland primary forests (Taiwan Forestry Bureau 2011). The least disturbed lowland tropical primary forest is confined to Nanjenshan Reserve which is in southern Taiwan (Editorial Committee of the Flora of Taiwan 1994–2003). In contrast, lower montane-lowland secondary forests constitute a high percentage (11.0% or 179,079.74 ha) of the forested area (Taiwan Forestry Bureau 2011).

Lower montane-lowland secondary forests situated in Meinong, southern Taiwan, are facing issues about conserving natural biodiversity and heritages. This area is a popular tourist spot and famous for two natural heritages: one is Yellow Butterfly Valley (YBV) and Shuanghsi Tropical Botanical Garden (STBG). The two natural heritages are dominated by exotic trees due to historical human management practices. YBV is a sightseeing spot for yellow butterflies (*Catopsilia pomona*) which are attracted by exotic Kassod tree (*Senna siamea*) timber plantations (Chen 2013). STBG is an arboretum which was established during the Japanese colonial period with diverse exotic species (Yang et al. 2010). The ongoing conservation conflict between the government and local Hakka community (a subgroup of the Han Chinese) is due to their protest against a reservoir construction plan since the early 1990s (Tsai et al. 2013). Local people aim for some possible alternatives to conserve the two natural heritages, along with their unique Hakka culture (Tsai et al. 2013). Although both natural heritages have been heavily modified by human activities, their original management practices have been abandoned for more than 20 years since 1992. The two abandoned human-modified forests are likely to have some degree of natural regeneration (Yang et al. 2010), and thus have positive ecological impacts. However, it is not clear what is their current regeneration status, what kinds of conservation actions can take and what kinds of management steps should be enforced. Therefore, it is vital to evaluate the regeneration status and ecological impacts of the two forests before making any conservation plans.

The aim of this research is to evaluate the regeneration status, in terms of forest structure, diversity, and composition, of the two human-modified forests in Meinong, Taiwan. One is a plantation with low richness in exotic tree species, and the other is an arboretum with high richness in exotic tree species. We select the remaining lowland tropical primary forest in the Nanjenshan Reserve as a reference plot. We ask (1) Is there a structural difference among the three forests? (2) What are the proportions of native saplings in the human-modified forests? (3) Are the introduced exotic species able to naturalize? We will then base on our findings to discuss the values and management plans for the human-modified forests, including the important but absent native tree species, the conservation values of naturally regenerated native tree species, and the invasive potential of non-native tree species.

## Methods

### Study plots

**Fig. 1:**
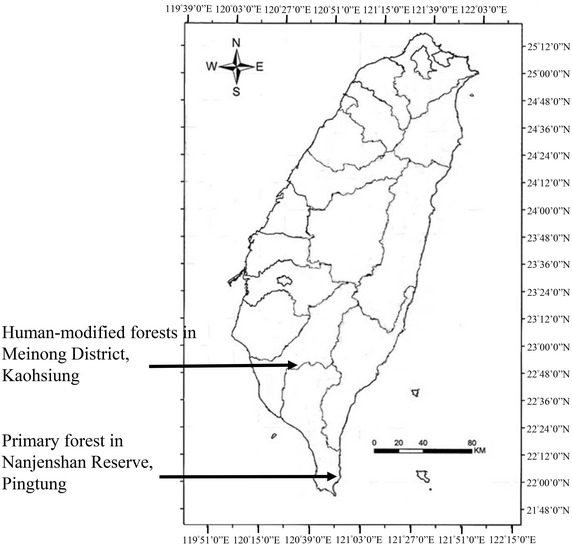
Location map of studied tropical human-modified forests and a reference forest in Taiwan. Nanjenshan Plot I (NPI) (the reference primary forest plot) is located in the Nanjenshan Reserve, Pingtung. Yellow Butterfly Valley (YBV) and Shuanghsi Tropical Botanical Garden (STBG) (the studied human-modified forest plots) are located in Meinong District, Kaohsiung

We chose two human-modified forests in Meinong District in Kaohsiung City, and one reference plot in the Nanjenshan Reserve in Pingtung County (Fig. 1). Both human-modified forests experienced negligible human disturbances since 1992. The reference primary forest, Nanjenshan Reserve, has been designated as a nature reserve since 1982.

The mean temperature in Meinong District is 24.0 °C (Central Weather Bureau 2014). The rainy season occurs from May to September. During this period, about 89% of the annual rainfall accumulated in the area. The lowland human-modified forests surrounding Meinong (295–650 m a.s.l.) are ecological buffer zones between human development and montane primary forests in the Central Mountain Range. In addition, mountains surrounding Meinong District shelter the human-disturbed forests from typhoon disturbances. Thus, exotic tropical timber species, such as those in the family of Dipterocarpaceae, were able to reach their natural heights of more than 30 m.

The first human-modified forest is an exotic species-poor plantation, Yellow Butterfly Valley (YBV; 120°35′33″E, 22°55′58″N). The area of YBV is 2805 ha (Chen 2013). YBV is a plantation where approximately 6 non-native timber and fruit tree species have been widely planted. Thus, the forest is relatively poor in its richness of non-native tree species. The name Yellow Butterfly Valley (YBV) is adopted by the local people referring to the yellow butterflies (*Catopsilia pomona*) which are attracted by the widely planted exotic tree, *Senna siamea* (Chen 2013). Based on our interviews with local farmers and researchers, YBV has experienced two intensive tree planting events. The first intensive period (from 1936 to 1945) was during the Japanese occupation. Two non-native timber trees, namely *Senna siamea,* and *Tectona grandis*, were extensively planted. The second intensive planting period was from 1949 to 1975, when the Taiwan Forestry Bureau enforced the rent afforestation policy. Fruit trees, including *Mangifera indica*, *Litchi chinensis*, *Euphoria longana*, and *Bambusa* spp., were commonly planted for afforestation and non-timber forest products. Based on land use policy amendment (Huang 2002) and local interviewees, logging activities eased around the late 1970s, reflecting the alteration of local economic activity. In 1992, some parts of YBV had additional plantations of *Mangifera indica* in order to receive governmental compensation for the Meinong reservoir plan (Meinung People’s Association 1994; Tsai et al. 2013). These *Mangifera* trees were not managed after planting because the reservoir construction plan was stopped by local people (Meinung People’s Association 1994). Thereafter, YBV has become a natural sightseeing attraction, especially for its yellow butterflies, with negligible human logging disturbances.

The other human-modified forest is an exotic species-rich arboretum, Shuanghsi Tropical Botanical Garden (STBG; 120°36′3″E, 22°56′10″N). STBG is adjacent to YBV and its area is 7.56 ha (Yang et al. 2010). STBG was originally known as the Meinong Twin Creek Arboretum which has high richness in exotic tree species. In 1935, 270 tree species were planted for the purpose of cultivating tropical non-native forest timber species (Yang et al. 2010). Chang (1970) found that majority of the originally planted species were not able to adapt to the environment in STBG, and by 1968 only 97 non-native species survived (Chang 1970). In 2008, the recorded non-native species in STBG was even lower, with only 75 non-native tree species (Yang et al. 2010). In 2008, the dominant (based on basal areas) non-native species in STBG included *Swietenia macrophylla*, *Spathodea nilotica* and *Terminalia calamnsanai* (Yang et al. 2010). Concurrently, more than 46 naturally recruited native tree species have been recorded, such as *Schefflera octophylla*, *Ficus irisana,* and *Machilus zuihoensis* (Yang et al. 2010). STBG was officially opened to public visitation in 1987 (Chuang 2013), and since then it has been jointly managed as a recreation park by the Taiwan Forestry Bureau and the local community (Chuang 2013).

The reference plot is within the Nanjenshan Reserve, namely Nanjenshan Plot I (NPI; 120°50′51″E, 22°04′54″N) (Fig. 1). Its elevation ranges from 224 to 275 m (Chao et al. 2010a). NPI was established in 1993 (2.1 ha in size) for the purpose of long-term ecological research (Chao et al. 2010a). The mean annual temperature is 22.7 °C and the mean annual rainfall is 3252 mm, without a dry season (Chao et al. 2010b). Tree census of NPI has been conducted every 7–9 years since its establishment (Chao et al. 2010a). The forest in NPI is a tropical lowland rainforest which was classified as a *Ficus*-*Machilus* zone (Hsieh et al. 2000). A more recent study categorizes the NPI forest as a tropical foothill evergreen broad-leaved forest, which is in a *Dysoxylum*–*Machilus* zone (Li et al. 2013). The major difference is that the *Ficus*–*Machilus* zone describes the overall lowland forests in Taiwan (Su 1984), whereas the *Dysoxylum*–*Machilus* zone is only the lowland forests confined to southern Taiwan (to the south of 23.2°N) (Li et al. 2013).

The reference primary forest is the least disturbed lowland tropical primary forest in Taiwan (Editorial Committee of the Flora of Taiwan 1994–2003), and it has long-term census records (Chao et al. 2010a). However, the reference forest is not a perfect control because it is located approximately 130 km away from the studied human-modified forests, and it has no dry season. Nevertheless, we adopted NPI as a reference forest for the following reasons: (1) There was no lowland primary forest in Meinong (Weng 2013). (2) Based on the vegetation classification scheme in Li et al. (2013), the potential tropical foothill evergreen broad-leaved forest in Taiwan is to the south of 23.2°N. Both Meinong District (22.9°N) and the Nanjenshan Reserve (22.1°N) are within the range. (3) All the three plots are relatively sheltered from the impacts of typhoon [a crucial disturbance type for vegetation in Taiwan (Lin et al. 2010)]. Therefore, although the reference plot was not perfect, it is able to give some insights into the forest structure and composition of potential primary forests. We did not intend to imply that the Nanjenshan forests would be the final successional forest for the two human-modified forests. Rather, the reference plot was used to provide a basal line for the comparison of diversity and regeneration status between the two human-modified forests.

### Quadrat sampling

For each of the human-modified forest, 4 transects were set up in July 2013. The distance between transects was at least 90 m and each transect had 4 quadrats. Therefore, a total of 16 quadrats (each size 10 m × 5 m) were sampled in each of the study plots (Loo 2015). In order to minimize environmental variations, we placed transects with aspects of approximately 300° and slope angles <40°. The quadrats were set up along a human accessibility distance gradient (from trail/river side into forest interior) at 10 m intervals. Some quadrats (n = 5 out of 32) did not follow the 10 m interval rule as we attempted to avoid steep slopes (>40°), hill top, and forest light gaps. These heterogenetic microenvironments were not considered in our study (Loo 2015).

In the Nanjenshan primary forest, we selected 16 quadrats (at 10–15 m intervals), measuring 10 m × 5 m. These quadrats were confined to those located in aspects at approximately 300° and slope angles <40° in the Nanjenshan Plot I (Loo 2015).

### Data collection

We conducted tree censuses of the human-modified forests in July and August 2013. We used tree census data of NPI conducted in 2008. Vegetation data, including tree species, height, and DBH (diameter at breast height), were collected for evaluating forest structure, diversity, and composition. All trees ≥1 cm DBH in the selected quadrats were measured. Plant identification and nomenclature were based on Flora of Taiwan (Editorial Committee of the Flora of Taiwan 1994–2003) with three exceptions. (1) *Radermachera sinica* was misspelled as *Radermachia sinica* in Li (1998). Our study used its correct name according to International Plant Names Index (2005). (2) *Glochidion ovalifolium* was not recorded in Flora of Taiwan. We followed the name of this species in Lu and Hsu (2003) and Hsu et al. (2006). (3) The familial classification of the recorded species were updated with APG IV system (The Angiosperm Phylogeny Group 2016).

### Data analysis

#### Forest structure

We classified tree heights into four classes: class 1: ≤5 m; class 2: >5 and ≤ 10 m; class 3: >10 and ≤20 m; class 4: >20 m. We also classified DBH measurements into three size classes: class 1: ≥1 and <10 cm; class 2: ≥10 and <20 cm; class 3: ≥20 cm. We assumed that trees in the size class 1 (DBH ≥1 and <10 cm) had naturally regenerated within 20 years, approximately after the major historical human disturbance. We assumed that trees in the size class 2 (DBH ≥10 and <20 cm) had survived for approximately 20–40 years. Also, we assumed that trees in the size class 3 (DBH ≥20 cm) were the remnant trees or planted trees that had survived for more than 40 years. We then compared the structures between the primary forest (i.e., NPI) and the human-modified forests (i.e., YBV and STBG) by the distribution frequency of height and size classes. We used the proportion of native and endemic species individuals to compare regeneration statuses in the three forests. We assumed that a higher percentage of sapling individuals belonging to species native to Taiwan would indicate a better regeneration status.

#### Species diversity

Species richness, effective species number, evenness, and rarefaction (Magurran 2004) were used to compare species diversity. Species richness (*S*) refers simply to the number of species, which is weighted by the number of rare species (Hill 1973). Effective species number (denoted by *N*_*1*_) is the exponentially transformed Shannon index (exp (*H*ʹ)) (Hill 1973).

$$N_{ 1} = { \exp }\left( { - \varSigma p_{i} { \ln }p_{i} } \right),$$

where *ρ*_*i*_ refers to the ratio of the individuals counted for the *i*_th_ species to the total individuals in a plot. Effective species number represents a diversity index weighted by the proportion of individuals. Evenness is *N*_1_/S which represents the equitability of the species in a community (Buzas and Hayek 1996). Due to differences in sampling efforts (e.g., the number of individuals) between communities, comparing rarefaction curves can help to account for the sampling efforts on the patterns of species diversity (Gotelli and Colwell 2001; Magurran 2004). The rarefaction curve is produced by randomly and repeatedly re-sampling a pool of *N* individuals or *N* quadrats, and then plotting the average number of species represented by 1 to *N* number of individuals or quadrats (Gotelli and Colwell 2001). All the indices were computed by the software PAST (PAleontological STatistics, version 3.04, Natural History Museum, University of Oslo, Norway).

#### Species importance values

We calculated the species importance values (IV %) as

$${\text{IV}}\% = \left( {RBA\% + RD\% + RF\% } \right)/ 3,{\text{ where}}$$

*RBA*% is a relative basal area, *RD*% is a relative density, and *RF* % is a relative frequency (Mueller-Dombois 1974). The equations are as follows:

$$\begin{aligned} RBA\% = \left( {BA/\varSigma BA{\text{of all the species in the plot}}} \right) \times 100\% ; \hfill \\ RD\% = \left( {D/\varSigma D{\text{of all the species in the plot}}} \right) \times 100\% ; \hfill \\ RF\% = \left( {F/\varSigma F{\text{of all the species in the plot}}} \right) \times 100\% ; \hfill \\ \end{aligned}$$

Basal area of an individual was calculated as π (DBH/2)^2^, and the values of all the individuals for a target species were summed and transformed into a unit area value (BA, m^2^ ha^−1^). Density (*D*, number of individuals ha^−1^) was the number of individuals per ha for a target species. Frequency (*F*) was the number of quadrats in which a target species was found within a plot, divided by the total number of quadrats in that plot (Mueller-Dombois 1974).

#### Similarity indices

We compared species similarities of the three plots using a qualitative similarity index of Sørensen and a quantitative similarity index of Motyka (Mueller-Dombois 1974). The Sørensen and Motyka indices emphasize different properties in similarity. The first emphasizes the presence or absence of a species. The latter considers the quantity of a species (e.g., density). The Sørensen similarity index *S*_s_ was calculated as

$$S_{s} = ( 2C/\left( {A + B} \right) \times 100\% ),$$

where *C* is the number of shared taxa found in both plots; *A* is the number of all taxa found in plot A; *B* is the number of all taxa found in plot B (Mueller-Dombois 1974). The Motyka similarity index *IS*_mo_ was calculated as

$$IS_{mo} = ( 2M_{w} /\left( {M_{A} + M_{B} } \right) \times 100\% ),$$

where *M*_*W*_ is the lower stem density of shared taxa found in both plots; *M*_*A*_ is the total stem density found in plot A; *M*_*B*_ is the total stem density found in plot B (Mueller-Dombois 1974). The possible maximum number of the two indices is 100%, indicating that the species composition of the compared plots is exactly the same.

## Results

We recorded a total of 1316 individuals from 88 species, belonging to 69 genera, and 34 families, in all three study plots. Among the recorded species, 73 species (including one variety *Litsea akoensis* var. *chitouchiaoensis*) were native to Taiwan, and 15 taxa were endemic to Taiwan (Additional file 1: Appendix A). We also recorded 15 species which were not native to Taiwan (Additional file 1: Appendix B). Notably, two recorded rare and native taxa (*Litsea akoensis* var. *chitouchiaoensis* and *Glochidion ovalifolium*) were both in YBV and STBG but not in NPI. The two species are endemic to the central and southern Taiwan (Editorial Committee of the Flora of Taiwan 1994–2003; Hsu et al. 2006). A total of 11 native species (taxa) were shared between the three plots (Additional file 1: Appendix A).

### Forest structure

**Fig. 2:**
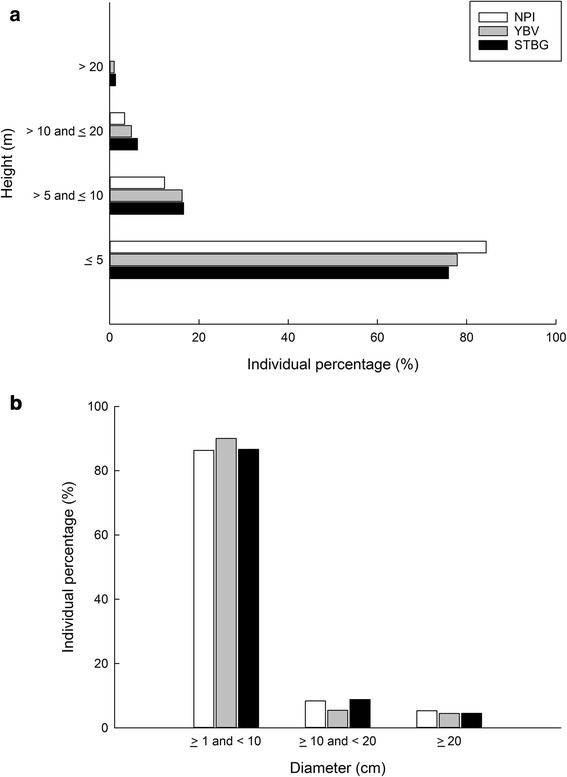
Forest structure of two tropical human-modified forest plots and the reference plot. The figures represent two structural indices: **a** height class and **b** diameter class. The height classes include class 1: ≤5 m; class 2: >5 and ≤10 m; class 3: >10 and ≤20 m; class 4: >20 m. Note that there was no individual in the height class 4 (>20 m) in NPI. The DBH (diameter at breast height) size class include class 1: ≥1 and <10 cm; class 2: ≥10 and < 20 cm; class 3: ≥20 cm. Abbreviations of study plots please refer to Fig. 1

Forest structures (frequency of height and size classes) of the three plots were quite similar (Fig. 2). However, overall stem densities were quite high in the human-modified forest plots (6162.5 and 5812.5 individual ha^−1^; Table 1). The maximum canopy height in STBG was the tallest (30 m), while the maximum canopy height in NPI was the shortest (13.5 m). The high canopy height in STBG was attributed to the non-native timber species at the height class 4 (height >20 m), including *Hevea brasiliensis, Sindora cochinchinensis*, *Swietenia macrophylla,* and *Tectona grandis*. Notably, the quadrats in the primary forest (NPI) did not have any trees at the height class 4 (Fig. 2a). Although NPI had the shortest maximum canopy height, it had the largest tree size among the three forests (DBH = 121 cm, *Bischofia javanica*).

**Table 1:** Forest structure and diversity indices of the human-modified forests and reference plot

Indices	NPI	YBV	STBG
Basal area (m^2^ ha^−1^)	45.4	39.2	39.0
Density (stems ha^−1^)	4475.0	6162.5	5812.5
Density_saplings (stems ha^−1^)	3862.5	5550.0	5037.5
Density_native saplings (stems ha^−1^)	3862.5	4750.0	2525.0
Species richness	45	40	52
Species richness_saplings	39	38	47
Species richness_native saplings	39	33	34
Effective species number	22.7	22.0	22.8
Effective species number_saplings	19.3	20.4	19.9
Effective species number_native saplings	19.3	18.7	17.2
Evenness	0.50	0.55	0.44
Evenness_saplings	0.49	0.54	0.42
Evenness_native saplings	0.49	0.57	0.51

Indices based on species native to Taiwan were noted with “_native”. Indices based on DBH (diameter at breast height) size class 1: ≥1 and < 10 cm were denoted with “_saplings”. Abbreviations of study plots please refer to Fig. 1

**Fig. 3:**
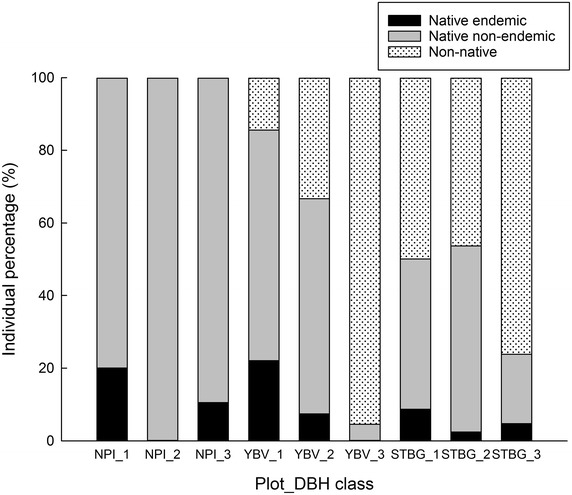
Forest regeneration quality of two tropical human-modified forest plots and the reference plot. The quality of forest regeneration is determined by the proportion of native and endemic species to Taiwan. Size class is the DBH (diameter at breast height) size class, including class 1: ≥1 and <10 cm; class 2: ≥10 and <20 cm; class 3: ≥20 cm. Abbreviations of study plots please refer to Fig. 1

All the study plots showed a similar proportion (86–90%) of saplings (size class 1; ≥1 and <10 cm; Fig. 2b). However, the proportion of native saplings was higher in YBV (86% out of 5550.0 sapling individuals ha^−1^) than in STBG (50% out of 5037.5 sapling individuals ha^−1^) (Fig. 3; Table 1), indicating a poor regeneration status of native sapling numbers in STBG.

### Species diversity

**Fig. 4:**
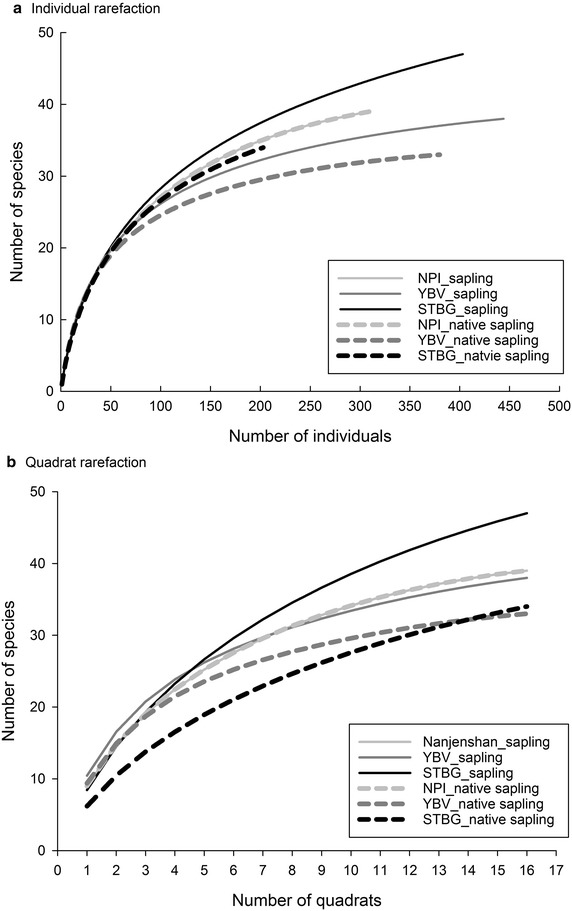
Rarefaction of two tropical human-modified forest plots and the reference plot. **a** Individual rarefaction and **b** quadrat rarefaction. *Solid lines* represent all the saplings, whereas the* dashed lines* represent saplings belonging to species native to Taiwan. In NPI, all the individuals are species native to Taiwan. Abbreviations of study plots please refer to Fig. 1

In general, species diversity indices were similar for all the three plots (Table 1). Notably, when sampling efforts of individual numbers were standardized, STBG had a higher average number of native saplings and steeper slopes of rarefaction curve than YBV (Fig. 4a). For quadrat rarefaction curve, STBG also had a steeper slope for native sapling species than YBV (Fig. 4b).

### Species importance values

The top 20 IV species accounted for 80.6, 88.5 and 78.9% of the total IV in NPI, YBV, and STBG, respectively (Table 2). The native species within the human-modified forests did not have similar ranks as those within the primary forest (Table 2). Only 11 out of the 45 native species in NPI (such as *Psychotria rubra*, *Lasianthus obliquinervis*, *Dendrocnide meyeniana*, *Melanolepis multiglandulosa*, and *Macaranga tanarius*) can also be found in the two human-modified forests (Table 2 and Additional file 1: Appendix A). Although some non-native species only had plantation records in STBG (no records in YBV), saplings of these species (such as *Spathodea nilotica* and *Markhamia hildebrandtii*) were found both in STBG and YBV (Additional file 1: Appendix B).

**Table 2:** The top 20 dominant species of two tropical human-modified forests and one reference plot

No.	Species name	Basal area (m^2^ ha^−1^)	Density (stems ha^−1^)	Frequency (%)	IV (%)
(a) Nanjenshan Plot I (NPI)					
1.	*Bischofia javanica*	15.0	25.0	12.5	11.6
2.	***Psychotria rubra***	0.5	600.0	68.8	7.0
3.	***Lasianthus obliquinervis***	0.1	487.5	81.3	6.2
4.	*Turpinia ternata*	1.6	362.5	75.0	6.2
5.	*Dysoxylum hongkongense*	3.1	262.5	56.3	6.0
6.	***Dendrocnide meyeniana***	1.9	212.5	68.8	5.1
7.	Strobilanthes longespicatus*	0.1	537.5	31.3	5.0
8.	*Aglaia elliptifolia*	0.3	312.5	62.5	4.5
9.	*Michelia compressa*	4.0	25.0	6.3	3.3
10.	*Beilschmiedia erythrophloia*	1.8	100.0	31.3	3.1
11.	*Lagerstroemia subcostata*	3.1	37.5	12.5	3.0
12.	Reevesia formosana*	3.1	37.5	12.5	3.0
13.	*Leea guineensis*	0.1	137.5	56.3	2.8
14.	*Schefflera octophylla*	1.8	75.0	18.8	2.5
15.	Drypetes karapinensis*	0.1	100.0	43.8	2.2
16.	*Ardisia sieboldii*	1.6	37.5	18.8	2.0
17.	***Melanolepis multiglandulosa***	0.1	137.5	31.3	2.0
18.	*Glycosmis citrifolia*	0.1	100.0	37.5	2.0
19.	*Cryptocarya concinna*	0.1	87.5	31.3	1.7
20.	***Macaranga tanarius***	0.4	62.5	25.0	1.5
	Subtotal of the top 20	39.1	3737.5	781.3	80.6
	Other species	6.3	737.5	300.0	19.4
	Total in NPI	45.4	4475.0	1081.3	100.0
(b) Yellow Butterfly Valley (YBV)					
1.	*Senna siamea* ^#^	28.5	325.0	68.8	28.0
2.	*Champereia manillana*	1.2	800.0	100.0	8.2
3.	*Mangifera indica* ^#^	2.0	700.0	68.8	7.5
4.	***Melanolepis multiglandulosa***	0.9	412.5	75.0	5.1
5.	*Litsea akoensis* var. chitouchiaoensis*	0.3	562.5	50.0	4.7
6.	***Psychotria rubra***	0.1	562.5	43.8	4.4
7.	*Glochidion philippicum*	0.8	237.5	62.5	3.7
8.	*Wendlandia uvariifolia*	0.7	175.0	43.8	2.7
9.	*Machilus japonica* var. kusanoi*	0.2	225.0	43.8	2.6
10.	*Ardisia cornudentata* subsp. *morrisonensis*	0.0	237.5	43.8	2.6
11.	***Ficus septica***	0.4	162.5	43.8	2.5
12.	Glochidion ovalifolium*	0.3	175.0	43.8	2.4
13.	***Dendrocnide meyeniana***	0.5	137.5	37.5	2.2
14.	***Ficus ampelas***	0.4	137.5	31.3	2.0
15.	Lindera akoensis*	0.0	125.0	43.8	1.9
16.	***Melicope semecarpifolia***	0.3	137.5	31.3	1.9
17.	Litsea hypophaea*	0.2	100.0	31.3	1.6
18.	*Bridelia tomentosa*	0.1	125.0	31.3	1.6
19.	*Murraya paniculata*	0.0	75.0	37.5	1.5
20.	*Ficus irisana*	0.5	75.0	18.8	1.4
	Subtotal of the top 20	37.5	5487.5	950.0	88.5
	Other species	1.7	675.0	225.0	11.5
	Total in YBV	39.2	6162.5	1175.0	100.0
(c) Shuanghsi Tropical Botanical Garden (STBG)					
1.	*Sindora cochinchinensis* ^#^	10.5	1175.0	18.8	16.3
2.	*Wendlandia uvariifolia*	1.6	625.0	87.5	7.9
3.	*Spathodea nilotica* ^#^	2.7	300.0	50.0	5.7
4.	*Swietenia macrophylla* ^#^	2.1	337.5	37.5	5.0
5.	*Tectona grandis* ^#^	4.3	75.0	25.0	4.9
6.	*Champereia manillana*	0.3	387.5	62.5	4.6
7.	*Markhamia hildebrandtii* ^#^	0.6	500.0	31.3	4.4
8.	*Senna siamea* ^#^	3.3	37.5	12.5	3.5
9.	***Ficus ampelas***	0.5	150.0	56.3	3.1
10.	Glochidion ovalifolium*	1.2	175.0	31.3	3.1
11.	*Litsea akoensis* var. chitouchiaoensis*	0.1	200.0	50.0	2.9
12.	*Hevea brasiliensis* ^#^	1.4	150.0	18.8	2.7
13.	***Ficus septica***	1.1	112.5	31.3	2.6
14.	*Litsea perrottetii* ^#^	1.3	137.5	12.5	2.3
15.	*Terminalia calamansanai* ^#^	0.9	62.5	25.0	1.9
16.	*Glochidion philippicum*	0.3	112.5	25.0	1.7
17.	*Murraya paniculata*	0.2	100.0	25.0	1.6
18.	***Macaranga tanarius***	0.4	62.5	25.0	1.6
19.	***Dendrocnide meyeniana***	0.4	62.5	25.0	1.5
20.	***Melicope semecarpifolia***	0.3	62.5	25.0	1.5
	Subtotal of the top 20	33.5	4825.0	675.0	78.9
	Other species	5.5	987.5	318.8	21.1
	Total in STBG	39.0	5812.5	993.8	100.0

Species rank is based on the importance values (IV%). (a) Nanjenshan Plot I (NPI) is a reference primary forest plot. (b) Yellow Butterfly Valley (YBV) and (c) Shuanghsi Tropical Botanical Garden (STBG) are human-modified forest plots

* Endemic species; ^#^ exotic species; species which are shared between the three plots are marked in bold italic (see also Additional file 1: Appendix A)

### Similarity indices

Compared STBG with YBV, STBG had relatively high similarities to NPI in terms of the presence and absence of native sapling species (*S*_*s*_: 38%; Table 3), but not in terms of quantity (*IS*_*mo*_: 13%; Table 3). Meanwhile, the similarity between STBG and YBV was high for the presence of shared native sapling species (*S*_*s*_: 75%; Table 3), but not in terms of their quantity of shared native sapling species (*IS*_*mo*_: 49%; Table 3).

**Table 3:** Species similarity indices of two tropical human-modified forests and one reference plot

	All individuals		Saplings		Native saplings	
	*S*_*s*_ (%)	*IS*_*mo*_ (%)	*S*_*s*_ (%)	*IS*_*mo*_ (%)	*S*_*s*_ (%)	*IS*_*mo*_ (%)
YBV & NPI	31	21	29	22	33	24
STBG & NPI	33	12	30	10	38	13
YBV & STBG	67	37	66	36	75	49

*S*_*s*_ is the Sørensen index and *IS*_*mo*_ is the Motyka index. Three data groups (all individuals, saplings, and native saplings) were used for the similarity comparison. Abbreviations of study plots please refer to Fig. 1

## Discussion

This study demonstrated the regeneration status and conservation values of two human-modified forests. Marcano-Vega et al. (2002) suggested that after abandonment for more than 30 years, human-modified lands are able to converge in forest structure, biodiversity, and composition, regardless of historical land use types (Marcano-Vega et al. 2002). Our study supports partly of the hypothesis, such that the two human-modified forests have similar forest structure (Fig. 2), diversity indices (Table 1), and qualitative species similarity (*S*_*s*_ = 75% in Table 3). However, the species diversity characterized by rarefaction curves and quantitative species similarity differed between the two human-modified forests. STBG (arboretum; rich in exotic tree species) seemed to have less density (Table 1) and proportion (Fig. 3) of native saplings than YBV (plantation; poor in exotic tree species). Nonetheless, STBG did have the potential to harbor more native species than YBV as estimated by the rarefaction curves (Fig. 4).

In the following sections, we discuss future management and conservation issues for the human-modified forests. Our major aspects of concerns are (1) the regeneration status in the human-modified forests; (2) conservation plans for the native species; (3) management plans for the non-native species.

### Structure, diversity, and composition status

The structure of the two human-modified forests was comparable to that of the primary forest. However, their species compositions, especially in terms of quantity (Table 3), were not. As such, comparing just the forest structure may result in a misleading conclusion about forest naturalness, as mature secondary forests and primary forests are indistinguishable by structure (Richards 1952; Chazdon 2014). However, compared with the primary forest plot, the human-modified forest plots had high stem densities (Table 1), suggesting that they were both in a developing stage. Their reverse J-shaped distribution of diameter classes (Fig. 2b) signified the potential of the human-modified forests to have a certain degree of natural regeneration.

Although the diversity indices seemed similar between the two human-modified forests (Table 1), patterns of rarefaction curves were different. In the 1970s, the diversities of non-native overstorey tree species differed for the two human-modified forests (about 97 exotic species in STBG and 6 exotic species in YBV). Even so, the two forests are adjacent to each other, and thus the capacity of native species pool should be similar. After abandoning the original management practices and allowing free competition for more than 20 years, their composition was converged in terms of the quality of native saplings species (*S*_*s*_ = 75%), but not in quantity (*IS*_*mo*_ = 49%) (Table 3). Notably, species richness of native saplings at STBG for an equivalent number of individuals is higher and with a steeper slope than that of YBV (Fig. 4a). The results demonstrated the potential for STBG to harbor more native species than YBV. In contrast, the overall richness of YBV leveled off faster than STBG (Fig. 4b), showing high species evenness but low potential to harbor more native species. Therefore, we propose that a species-rich plantation has a potential to harbor more native species if allowing more native individuals to grow.

The differences in native sapling diversity patterns are likely to be due to their original overstorey species composition (Parrotta 1995; Heinrichs et al. 2016). Previous studies have found that some exotic overstorey trees may hinder the regeneration of native saplings, whereas some may catalyze it (e.g., Parrotta 1995). Although we currently do not know which exotic species in our study forests had positive or negative effects on native sapling regenerations, we propose that the original overstorey diversity can be a crucial factor for natural regeneration. This is compared with the possible influences of a monoculture exotic species plantation. In a monoculture exotic species plantation, its effects on native saplings would be relatively monotonic, either negative (inhibition or competition), neutral, or positive (facilitation). However, in an exotic species-rich plantation, the overall effect is a combination of any possible inhibition, competition, or facilitation effects from the overstorey composition. Thus, it is likely that at the forest-level, a higher diversity of native saplings can be observed in a species-rich plantation than in a species-poor plantation. In other words, a diverse species overstorey (such as arboreta and agroforestry sites) may be able to support and nurse more native species.

Although our sample size is not large, we found that all the curves had leveled off (Fig. 4). This means that we have reached an asymptotic richness and dominant species have been sampled (Gotelli and Colwell 2001). Moreover, the top 20 species accounted for more than 78% of the IV (Table 2), suggesting that those non-recorded species are likely to be rare species. For example, some native species in YBV and STBG were not recorded in the selected quadrats in NPI but they were recorded in the census data in 2000 and in 2008 in the Nanjenshan Plot I. These species are: *Machilus japonica* var. *kusanoi*, *Machilus zuihoensis*, *Ficus irisana*, *Ficus superba*, *Ficus virgata*, *Morus australis*, and *Champereia manillana*. They all have low relative density in Nanjenshan Plot I (<1% relative density (RD); Chao 2001; Chao et al. 2010a). Thus, a small sample size can influence the composition results, but such an influence would be mainly on rare species.

### Management implications for native species

It is important for managers to be aware of the regeneration status of native species in human-modified forests as this determines the structure and composition of future communities (Chazdon 2014). The recruitment of native species in human-modified forests are likely to be from the surrounding remnant secondary forests, or the dispersal of seeds from other areas by wind or small animals (Parrotta 1995). Chazdon (1998) proposes that human-modified forests can be a biodiversity cornucopia. The on-going regeneration of native species found in the two human-modified forests supports the concept. Our results show that both YBV and STBG harbored species of the *Ficus*-*Machilus* vegetation type, which was once widespread in the lowland areas of Taiwan (Editorial Committee of the Flora of Taiwan 1994–2003). The diagnostic species for lowland tropical forests of Taiwan (Chiou et al. 2009; Li et al. 2013) can also be found in the two human-modified forests, including *Dendrocnide meyeniana, Champereia manillana, Bischofia javanica*, and *Ficus irisana*. The results demonstrate that these human-modified forests are acting as refuges for the remnant native biodiversity.

Remarkably, two taxa (*Litsea akoensis* var. *chitouchiaoensis* and *Glochidion ovalifolium*) recorded in YBV and STBG are rare and endemic species to central and southern Taiwan (Editorial Committee of the Flora of Taiwan 1994–2003; Hsu et al. 2006). The type specimen of *Litsea akoensis* var. *chitouchiaoensis* was collected in Chitouchiao village, which is adjacent to STBG and YBV (Liao 1982; Editorial Committee of the Flora of Taiwan 1994–2003). *Glochidion ovalifolium* was recorded only in the Chiayi County (Hsu et al. 2006), and its southern distribution in YBV was first documented by our study. These findings further support the conservation values of human-modified forests as biodiversity refuges, especially when primary forests are not available in the surrounding locality.

Although forests of YBV and STBG may not develop into the same type of forests as NPI, the comparison between the primary forest and the human-modified forests could provide some insights into conservation decision making. For example, the density of native saplings is still relatively low in STBG (Table 1). As there are no remnant primary forests within close proximity to Meinong (Weng 2013), it is critical to develop a list of species suitable for reintroduction to the area. After examining the species composition in NPI and their distribution range in Taiwan (based on Editorial Committee of the Flora of Taiwan 1994–2003), we have identified some possible native species for reintroduction. For example, we can introduce *Michelia compressa*, *Ardisia sieboldii*, and *Ilex rotunda* (the dominant species listed in Chiou et al. 2009; Li et al. 2013) to assist the recovery of YBV and STBG forests toward primary species composition. Other species, such as *Dysoxylum hongkongense* in NPI, are confined in the Hungchun Peninsula which would not be suitable for Meinong area. Without active management practices, the current dominance of some pioneers, such as *Melanolepis multiglandulosa*, *Ficus septica*, *Ficus ampelas*, and *Macaranga tanarius*, may persist in these human-modified forests.

### Management implications for non-native species

The heritage values of exotic tropical species in STBG and YBV could be maintained with caution. For example, yellow butterflies (*Catopsilia pomona*) in YBV are attracted to the exotic tree species, *Senna siamea* (Chen 2013). The butterfly is one of the important assets for the local people to develop tourism (Tsai et al. 2013). However, we did not find any naturally regenerated *Senna siamea* saplings. Hence, managers could try to plant *Senna siamea*. The absence of naturally regenerated saplings of *Senna siamea* in YBV and STBG may be attributed to water deficiency during the seedling establishment stage. Kondoh et al. (2006) suggest that *Senna siamea* has high water efficient vessels, resulting in a fast growth rate, but also making it more susceptible to drought. The fruiting season of *Senna siamea* coincides with the drought period, from October to April, in Meinong. Thus, seedlings were hardly recorded (Yang et al. 2010). Our follow up observation showed that *Senna siamea* seeds collected in YBV germinated and established after soaking with sufficient water (LCL personal observation). However, there has been no record of successful natural regeneration of *Senna siamea* since 1936 (Yang et al. 2010).

While acknowledging the heritage values of exotic tropical species in STBG and YBV, managers should also be aware of the invasive potential of some exotic tree species. Trees that have regenerated and naturalized in STBG may become invasive species to primary forests in the future. For example, *Sindora cochinchinensis*, *Markhamia hildebrandtii*, *Swietenia macrophylla*, *Spathodea nilotica,* and *Litsea perrottetii* had individuals in size class 1 (DBH ≥1 and <10 cm) (Additional file 1: Appendix B), indicating successful regeneration and possible naturalization of these species. Yang et al. (2010) noted an increase in population size of 38 non-native species in STBG, especially *Swietenia macrophylla*, *Spathodea nilotica*, and *Litsea perrottetii*. Extraordinarily, individuals of *Swietenia macrophylla* had increased from the initially planted 373 individuals in 1935–1257 individuals in 2008 (Yang 2001; Yang et al. 2010). Special caution is urgently required to manage these naturalized non-native species.

Our study further provides evidence of the first alert that two of the non-native species in STBG, *Spathodea nilotica* and *Markhamia hildebrandtii*, have saplings recorded in YBV (Additional file 1: Appendix B). Due to no records of adult tree plantation in YBV, the two species could have been dispersed naturally from STBG to YBV, suggesting an invasive potential. The observed pattern implied that an exotic species-rich plantation is likely to introduce some invasive species in the new locality, based on the tens-rule (Williamson et al. 1986; Richardson and Pyšek 2006). One of the two naturalized species, *Spathodea nilotica*, is from the same genus as *Spathodea campanulata*, which has already diagnosed as a highly invasive tree in the Pacific region (Pacific Island Ecosystem at Risk 2010). Moreover, *Spathodea nilotica* has winged seeds that are likely to be dispersed by wind. The second naturalized species, *Markhamia hildebrandtii* belongs to the same family as *Spathodea nilotica* (Bignoniaceae) and it also has winged seeds. For winged-seed > 300 mg, the travel distance can be up to 100 m (Corlett 2009). Therefore, these two wind-dispersed trees have the potential to migrate from the current location into other nearby forests. The potential of this negative ecological impact needs to be further monitored.

## Conclusions

We have compared and evaluated the regeneration status of two human-modified forests by studying their forest structure, diversity, and composition. After having been abandoned and allowing for natural regeneration for more than 20 years, the two human-modified forests are comparable to the primary forest in terms of forest structure. This similarity is regardless of prior to abandonment exotic species richness. However, they differ in detailed species composition and diversity patterns, suggesting the need to enforce some management practices.

We suggest that in order to keep the two heritages with high biodiversity values, management practices should focus on (1) providing seed or seedling sources of important but missing primary forest species (such as *Michelia compressa*, *Ardisia sieboldii*, and *Ilex rotunda*); (2) conserving the recorded rare and endemic taxa (*Litsea akoensis* var. *chitouchiaoensis* and *Glochidion ovalifolium*); (3) monitoring exotic species with invasive potential (such as *Spathodea nilotica* and *Markhamia hildebrandtii*). Our study supports the hypothesis that human-modified forests have the potential to be refuges for remnant biodiversity. Moreover, we propose that the diversity of exotic overstorey composition can possibly nurse the diversity of understorey sapling species together with the risk to introduce invasive species. The follow-up step would be to facilitate these human-modified forests into regeneration and conservation nuclei for future forests.

## Additional file

**Additional file 1:** **Appendix A**. Native species recorded in the study plots. **Appendix B**. Non-native species recorded in the study plots.

## Abbreviations

IV: importance values
NPI: Nanjenshan Plot I
STBG: Shuanghsi Tropical Botanical Garden
YBV: Yellow Butterfly Valley
