# Supplementary material for: Characteristics of tropical human-modified forests after 20 years of natural regeneration
Source: Bot Stud. 2017 Aug 30;58:36. doi: 10.1186/s40529-017-0190-x (PMC5578950; doi:10.1186/s40529-017-0190-x)
Supplement: Supplementary file 1 — Additional file 1: Appendix A. Native species recorded in the study plots. Appendix B. Non-native species recorded in the study plots. [file 40529_2017_190_MOESM1_ESM.docx]

Appendix A. Native species recorded in the study plots. The stem density (stems 0.08 ha^-1^) by size classes 1, 2 and 3 of the reference primary forest (Nanjenshan Plot I (NPI)) were compared with the two human-disturbed forests (Yellow Butterfly Valley (YBV) and Shuanghsi Tropical Botanical Garden (STBG)). Species denoted by * is endemic to Taiwan. Species marked in bold are shared between the three plots. Size class includes class 1: ≥ 1 cm and < 10 cm; class 2: ≥ 10 cm and < 20 cm; class 3: ≥ 20 cm. Plant identification and nomenclature were based on Flora of Taiwan ([Editorial Committee of the Flora of Taiwan 1994–2003](#_ENREF_16)), with familial classification updated with APG IV system ([The Angiosperm Phylogeny Group 2016](#_ENREF_48)).

|  |  | NPI | | | YBV | | | STBG | | |
| --- | --- | --- | --- | --- | --- | --- | --- | --- | --- | --- |
| Family | Species | 1 | 2 | 3 | 1 | 2 | 3 | 1 | 2 | 3 |
| Acanthaceae | *Strobilanthes longespicatus** | 43 | 0 | 0 | 0 | 0 | 0 | 0 | 0 | 0 |
| Adoxaceae | *Viburnum odoratissimum* | 2 | 0 | 0 | 0 | 0 | 0 | 0 | 0 | 0 |
| Aquifoliaceae | *Ilex asprella* | 0 | 0 | 0 | 3 | 0 | 0 | 0 | 0 | 0 |
| Aquifoliaceae | *Ilex rotunda* | 0 | 0 | 1 | 0 | 0 | 0 | 0 | 0 | 0 |
| Aquifoliaceae | *Ilex uraiensis* | 3 | 0 | 1 | 0 | 0 | 0 | 0 | 0 | 0 |
| Araliaceae | *Schefflera octophylla* | 3 | 1 | 2 | 0 | 0 | 0 | 4 | 2 | 0 |
| Bignoniaceae | *Radermachera sinica* | 0 | 0 | 1 | 0 | 0 | 0 | 0 | 0 | 0 |
| Boraginaceae | *Cordia aspera* subsp. *kanehirai* | 1 | 0 | 0 | 0 | 0 | 0 | 0 | 0 | 0 |
| Boraginaceae | *Ehretia acuminata* | 2 | 0 | 0 | 0 | 0 | 0 | 0 | 0 | 0 |
| Boraginaceae | *Trichodesma calycosum* | 0 | 0 | 0 | 0 | 0 | 0 | 2 | 0 | 0 |
| Cannabaceae | *Celtis formosana** | 2 | 0 | 0 | 0 | 0 | 0 | 2 | 0 | 0 |
| Cannabaceae | *Trema orientalis* | 5 | 0 | 0 | 0 | 0 | 0 | 0 | 1 | 0 |
| Capparaceae | *Crateva adansonii* subsp. *formosensis** | 0 | 0 | 1 | 0 | 0 | 0 | 0 | 0 | 0 |
| Ebenaceae | ***Diospyros eriantha*** | 0 | 1 | 0 | 3 | 0 | 0 | 1 | 0 | 0 |
| Elaeocarpaceae | *Sloanea formosana** | 1 | 0 | 0 | 0 | 0 | 0 | 0 | 0 | 0 |
| Euphorbiaceae | ***Macaranga tanarius*** | 3 | 2 | 0 | 2 | 2 | 0 | 3 | 2 | 0 |
| Euphorbiaceae | *Mallotus japonicus* | 0 | 0 | 0 | 1 | 0 | 0 | 0 | 0 | 0 |
| Euphorbiaceae | ***Mallotus paniculatus*** | 3 | 1 | 0 | 0 | 1 | 0 | 1 | 1 | 0 |
| Euphorbiaceae | ***Melanolepis multiglandulosa*** | 11 | 0 | 0 | 30 | 3 | 0 | 5 | 2 | 0 |
| Fabaceae | *Acacia confusa* | 0 | 0 | 0 | 0 | 0 | 0 | 0 | 0 | 1 |
| Fabaceae | *Archidendron lucidum* | 0 | 0 | 0 | 5 | 0 | 0 | 0 | 0 | 0 |
| Fagaceae | *Castanopsis indica* | 2 | 1 | 0 | 0 | 0 | 0 | 0 | 0 | 0 |
| Lamiaceae | *Callicarpa formosana** | 0 | 0 | 0 | 2 | 0 | 0 | 2 | 0 | 0 |
| Lamiaceae | *Callicarpa remotiserrulata** | 1 | 0 | 0 | 0 | 0 | 0 | 0 | 0 | 0 |
| Lamiaceae | *Clerodendrum kaempferi* | 0 | 0 | 0 | 0 | 0 | 0 | 2 | 0 | 0 |
| Lauraceae | *Beilschmiedia erythrophloia* | 7 | 0 | 1 | 0 | 0 | 0 | 0 | 0 | 0 |
| Lauraceae | *Cinnamomum kotoense* | 0 | 0 | 0 | 0 | 0 | 0 | 1 | 0 | 0 |
| Lauraceae | *Cryptocarya concinna* | 6 | 1 | 0 | 0 | 0 | 0 | 0 | 0 | 0 |
| Lauraceae | *Lindera akoensis** | 0 | 0 | 0 | 10 | 0 | 0 | 2 | 0 | 0 |
| Lauraceae | *Litsea akoensis* var. *akoensis** | 2 | 0 | 0 | 0 | 0 | 0 | 0 | 0 | 0 |
| Lauraceae | *Litsea akoensis* var. *chitouchiaoensis** | 0 | 0 | 0 | 45 | 0 | 0 | 16 | 0 | 0 |
| Lauraceae | *Litsea hypophaea** | 0 | 0 | 0 | 7 | 1 | 0 | 0 | 0 | 0 |
| Lauraceae | *Machilus japonica* var. *kusanoi** | 0 | 0 | 0 | 18 | 0 | 0 | 1 | 1 | 0 |
| Lauraceae | *Machilus zuihoensis** | 0 | 0 | 0 | 3 | 1 | 0 | 1 | 0 | 0 |
| Lythraceae | *Lagerstroemia subcostata* | 0 | 0 | 3 | 0 | 0 | 0 | 0 | 1 | 0 |
| Magnoliaceae | *Michelia compressa* | 1 | 0 | 1 | 0 | 0 | 0 | 0 | 0 | 0 |
| Malvaceae | *Reevesia formosana** | 2 | 0 | 1 | 0 | 0 | 0 | 0 | 0 | 0 |
| Meliaceae | *Aglaia elliptifolia* | 25 | 0 | 0 | 0 | 0 | 0 | 0 | 0 | 0 |
| Meliaceae | *Dysoxylum hongkongense* | 10 | 10 | 1 | 0 | 0 | 0 | 0 | 0 | 0 |
| Moraceae | ***Ficus ampelas*** | 2 | 0 | 0 | 9 | 2 | 0 | 10 | 2 | 0 |
| Moraceae | *Ficus fistulosa* | 3 | 0 | 0 | 0 | 0 | 0 | 0 | 0 | 0 |
| Moraceae | *Ficus irisana* | 0 | 0 | 0 | 4 | 2 | 0 | 0 | 0 | 1 |
| Moraceae | ***Ficus septica*** | 1 | 0 | 0 | 12 | 1 | 0 | 8 | 0 | 1 |
| Moraceae | *Ficus superba* | 0 | 0 | 0 | 0 | 0 | 0 | 1 | 0 | 0 |
| Moraceae | *Ficus virgata* | 0 | 0 | 0 | 2 | 0 | 0 | 5 | 0 | 0 |
| Moraceae | *Maclura cochinchinensis* | 0 | 0 | 0 | 0 | 0 | 0 | 1 | 0 | 0 |
| Moraceae | *Morus australis* | 0 | 0 | 0 | 2 | 0 | 0 | 1 | 0 | 0 |
| Nyctaginaceae | *Pisonia umbellifera* | 0 | 0 | 0 | 1 | 0 | 0 | 0 | 0 | 0 |
| Opiliaceae | *Champereia manillana* | 0 | 0 | 0 | 62 | 2 | 0 | 31 | 0 | 0 |
| Phyllanthaceae | *Bischofia javanica* | 0 | 0 | 2 | 0 | 0 | 0 | 0 | 0 | 0 |
| Phyllanthaceae | *Bridelia balansae* | 4 | 1 | 0 | 0 | 0 | 0 | 0 | 0 | 0 |
| Phyllanthaceae | *Bridelia tomentosa* | 0 | 0 | 0 | 10 | 0 | 0 | 3 | 1 | 0 |
| Phyllanthaceae | *Glochidion ovalifolium** | 0 | 0 | 0 | 14 | 0 | 0 | 13 | 0 | 1 |
| Phyllanthaceae | *Glochidion philippicum* | 0 | 0 | 0 | 18 | 0 | 1 | 8 | 1 | 0 |
| Primulaceae | *Ardisia cornudentata* subsp. *morrisonensis* | 0 | 0 | 0 | 19 | 0 | 0 | 7 | 0 | 0 |
| Primulaceae | *Ardisia sieboldii* | 1 | 1 | 1 | 0 | 0 | 0 | 0 | 0 | 0 |
| Primulaceae | *Ardisia virens* | 1 | 0 | 0 | 0 | 0 | 0 | 0 | 0 | 0 |
| Primulaceae | *Maesa perlaria* var. *formosana* | 0 | 0 | 0 | 4 | 0 | 0 | 0 | 0 | 0 |
| Putranjivaceae | *Drypetes karapinensis** | 8 | 0 | 0 | 0 | 0 | 0 | 0 | 0 | 0 |
| Rubiaceae | *Gardenia jasminoides* | 0 | 0 | 0 | 1 | 0 | 0 | 0 | 0 | 0 |
| Rubiaceae | *Lasianthus hiiranensis* | 4 | 0 | 0 | 0 | 0 | 0 | 0 | 0 | 0 |
| Rubiaceae | ***Lasianthus obliquinervis*** | 39 | 0 | 0 | 5 | 0 | 0 | 1 | 0 | 0 |
| Rubiaceae | *Lasianthus wallichii* | 3 | 0 | 0 | 0 | 0 | 0 | 0 | 0 | 0 |
| Rubiaceae | ***Psychotria rubra*** | 48 | 0 | 0 | 45 | 0 | 0 | 3 | 0 | 0 |
| Rubiaceae | *Tarenna gracilipes** | 3 | 0 | 0 | 1 | 0 | 0 | 0 | 0 | 0 |
| Rubiaceae | *Wendlandia uvariifolia* | 0 | 0 | 0 | 13 | 1 | 0 | 46 | 4 | 0 |
| Rutaceae | *Glycosmis citrifolia* | 8 | 0 | 0 | 1 | 0 | 0 | 0 | 0 | 0 |
| Rutaceae | ***Melicope semecarpifolia*** | 3 | 0 | 0 | 11 | 0 | 0 | 3 | 2 | 0 |
| Rutaceae | *Murraya paniculata* | 0 | 0 | 0 | 6 | 0 | 0 | 7 | 1 | 0 |
| Sapindaceae | *Sapindus mukorossii* | 1 | 1 | 1 | 2 | 1 | 0 | 2 | 1 | 0 |
| Staphyleaceae | *Turpinia ternata* | 22 | 7 | 0 | 0 | 0 | 0 | 0 | 0 | 0 |
| Urticaceae | ***Dendrocnide meyeniana*** | 12 | 3 | 2 | 10 | 1 | 0 | 4 | 0 | 1 |
| Vitaceae | *Leea guineensis* | 11 | 0 | 0 | 0 | 0 | 0 | 5 | 0 | 0 |
| Total |  | **309** | **30** | **19** | **381** | **18** | **1** | **202** | **22** | **5** |

Appendix B. Non-native species recorded in the study plots. The stem density (stems 0.08 ha^-1^) by size classes 1, 2 and 3 in the two human-modified forests, Shuanghsi Tropical Botanical Garden (STBG) and Yellow Butterfly Valley (YBV). Size class include class 1: DBH ≥ 1 cm and < 10 cm; class 2: ≥ 10 cm and < 20 cm; class 3: ≥ 20 cm.

|  |  | YBV |  |  | STBG | | |
| --- | --- | --- | --- | --- | --- | --- | --- |
| Family | Species | 1 | 2 | 3 | 1 | 2 | 3 |
| Anacardiaceae | *Mangifera indica* | 53 | 3 | 0 | 0 | 0 | 0 |
| Apocynaceae | *Alstonia scholaris* | 0 | 0 | 0 | 1 | 0 | 1 |
| Bignoniaceae | *Markhamia hildebrandtii* | 1 | 0 | 0 | 39 | 1 | 0 |
| Bignoniaceae | *Spathodea nilotica* | 5 | 1 | 0 | 16 | 7 | 1 |
| Combretaceae | *Terminalia calamansanai* | 0 | 0 | 0 | 3 | 1 | 1 |
| Elaeocarpaceae | *Elaeocarpus serratus* | 1 | 0 | 0 | 3 | 0 | 0 |
| Euphorbiaceae | *Aleurites trisperma* | 0 | 0 | 0 | 1 | 0 | 0 |
| Euphorbiaceae | *Hevea brasiliensis* | 0 | 0 | 0 | 11 | 0 | 1 |
| Fabaceae | *Albizia acle* | 0 | 0 | 0 | 1 | 0 | 0 |
| Fabaceae | *Senna siamea* | 0 | 5 | 21 | 0 | 0 | 3 |
| Fabaceae | *Sindora cochinchinensis* | 0 | 0 | 0 | 88 | 2 | 4 |
| Lamiaceae | *Tectona grandis* | 0 | 0 | 0 | 1 | 1 | 4 |
| Lauraceae | *Litsea perrottetii* | 0 | 0 | 0 | 7 | 4 | 0 |
| Meliaceae | *Swietenia macrophylla* | 0 | 0 | 0 | 23 | 3 | 1 |
| Sapindaceae | *Euphoria longana* | 4 | 0 | 0 | 6 | 0 | 0 |
| Total |  | 64 | 9 | 21 | 200 | 19 | 16 |
